# Female-germline specific protein Sakura interacts with Otu and is crucial for germline stem cell renewal and differentiation and oogenesis

**DOI:** 10.1101/2024.10.04.616675

**Published:** 2024-11-28

**Authors:** Azali Azlan, Li Zhu, Ryuya Fukunaga

## Abstract

During oogenesis, self-renewal and differentiation of germline stem cells (GSCs) must be tightly regulated. The *Drosophila* female germline serves as an excellent model for studying these regulatory mechanisms. Here, we report that a previously uncharacterized gene *CG14545* , which we named *sakura* , is essential for oogenesis and female fertility in *Drosophila* . Sakura is predominantly expressed in the ovaries, particularly in the germline cells, including GSCs. *sakura* null mutant female flies display rudimentary ovaries with germline-less and tumorous phenotypes, fail to produce eggs, and are completely sterile. The germline-specific depletion of *sakura* impairs Dpp/BMP signaling, leading to aberrant *bag-of-marbles* ( *bam* ) expression, resulting in faulty differentiation and loss of GSCs. Additionally, *sakura* is necessary for normal piwi-interacting RNAs (piRNAs) levels and for proper localization of Ool8 RNA-binding protein (Orb) in developing oocytes. We identified Ovarian Tumor (Otu) as protein binding partner of Sakura, and we found that loss of *otu* phenocopies loss of *sakura* in ovaries. Thus, we identified Sakura as a crucial factor for GSC renewal and differentiation and oogenesis, and propose that Sakura and Otu function together in these processes.

## Full Text Availability

The license terms selected by the author(s) for this preprint version do not permit archiving in PMC. The full text is available from the preprint server.

