# Supplementary material for: Female-germline specific protein Sakura interacts with Otu and is crucial for germline stem cell renewal and differentiation and oogenesis
Source: bioRxiv. 2025 Jun 4:2024.10.04.616675. Preprint. [Version 4] doi: 10.1101/2024.10.04.616675 (PMC11623502; doi:10.1101/2024.10.04.616675)

bioRxiv preprint doi: https://doi.org/10.1101/2024.10.04.616675; this version posted June 4, 2025. The copyright holder for this preprint (which was not certified by peer review) is the author/funder, who has granted bioRxiv a license to display the preprint in perpetuity. It is made available under aCC-BY 4.0 International license.

Fig S1. *sakura* mRNA expression pattern.

Data obtained from http://flybase.org/reports/FBgn0040602.htm.

Fig S2. Male fertility assay.

The numbers of the progeny flies obtained from crosses between test males and wild-type (OregonR) virgin females are shown. Mean ± SD (n = 5).

Fig S3. *sakura*^{null} ovaries are tumorous

bioRxiv preprint doi: https://doi.org/10.1101/2024.10.04.616675; this version posted June 4, 2025. The copyright holder for this preprint (which was not certified by peer review) is the author/funder, who has granted bioRxiv a license to display the preprint in perpetuity. It is made available under aCC-BY 4.0 International license.

Violin plots of GSC-like cell numbers in germaria of indicated genotypes of 2-5 days old flies. Mean ± SD and the biological replicate number n are also shown. P-value < 0.001 (Student's t-test, unpaired, two-tailed) is indicated by ***.

Fig S4. The ratio of germless ovarioles increases over time in *sakura*^{null} ovaries

(A) Ratio (%) of normal, germless, and tumorous ovarioles of the indicated genotypes at 0-1 days, 7 days, and 14 days post-eclosion. (B) Quantification of GSC-like cell numbers in ovarioles of the indicated genotypes of at 0-1 days, 7 days, and 14 days post-eclosion. Germless ovarioles were excluded from this analysis.

Fig S5. Sex-specific alternative splicing of *sxl* is dysregulated in *sakura*^{null} ovaries

Sex-specific alternative splicing of *sxl* was analyzed by RT-CPR followed by agarose gel electrophoresis and SYBR Safe staining. Ovaries from controls and *sakura-EGFP* rescue flies showed the expected female-specific *sxl* isoform, while testes from control flies exhibited the male-specific isoform. *sakura*^{null} ovaries showed increased expression of the male-specific isoform and reduced levels of female-specific isoform.

Fig S6. *sakura* is important for oogenesis in later-stage egg chambers

Confocal images of ovaries from *sakura* RNAi knockdown flies (*UAS-Dcr2*, *TOsk-Gal4* > *sakura*^{RNAi}) stained with phalloidin (F-Actin) and anti-Orb antibodies. F-Actin (red), Orb (green), and DAPI (blue). Yellow arrows indicate normal Orb enrichment at the posterior of developing

bioRxiv preprint doi: https://doi.org/10.1101/2024.10.04.616675; this version posted June 4, 2025. The copyright holder for this preprint (which was not certified by peer review) is the author/funder, who has granted bioRxiv a license to display the preprint in perpetuity. It is made available under aCC-BY 4.0 International license.

oocytes. White arrowheads indicate egg chambers exhibiting cytoskeletal disorganization. Scale bars: 50 μm.

Fig S7. *sakura*^{null} clone germline cells intrinsically cause tumorous phenotype

Number of GFP-positive GSC-like cells in germaria with marked (GFP-negative) GSCs of the indicated genotypes at 4, 7, and 14 days after clone induction. GSC-like cells containing round spectrosome were identified through immunostaining with anti-Hts antibody.

Fig S8. Bam is not misexpressed in TOsk-Gal4 > *sakura*^{RNAi} egg chambers.

Confocal images of ovaries from *sakura* RNAi knockdown flies driven by *UAS-Dcr2* and *TOsk-Gal4*, stained with anti-Bam and anti-DE-Cadherin (DE-Cad) antibodies. *y-RNAi* was used as a control. Bam (green), DE-Cad (red), and DAPI (blue). Cyan arrowheads indicate high Bam expression in 8-cell cysts within the germarium. White arrowheads indicate egg chambers with cytoskeletal disorganization. Scale bars: 50 μm.

Fig S9. Ratio of germless and tumorous phenotypes from double RNAi knockdown of *sakura* with *bam*, *cycA*, or *otu*

(A) Ratio (%) of normal, germless, and tumorous ovarioles in double RNAi knockdown of *sakura* with *bam, cycA,* or *otu. UAS-Dcr2* and *NGT-Gal4* was used to drive RNAi knockdown in the germline. n is the total number of ovarioles examined for the indicated genotypes of 2-5 day-old

bioRxiv preprint doi: https://doi.org/10.1101/2024.10.04.616675; this version posted June 4, 2025. The copyright holder for this preprint (which was not certified by peer review) is the author/funder, who has granted bioRxiv a license to display the preprint in perpetuity. It is made available under aCC-BY 4.0 International license.

flies. (B) Quantification of GSC-like cell number in germaria of indicated genotypes of 2-5 day-old flies. Germless ovarioles were excluded from this analysis.

Fig S10. *nos-Gal4-VP16 > UASp-Mad-GFP* did not rescue the *sakura*^{null} phenotyopes

(A) Ratio (%) of normal, germless, and tumorous ovarioles in the indicated genotypes of 2-5 day-old flies. n denotes the total number of ovarioles examined for each genotype. (B) Quantification of GSC-like cell number in germaria of the indicated genotypes from 2-5 day-old flies. Germless ovarioles were excluded from this analysis.

Fig S11. Ratio of germless and tumorous phenotypes in *sakura* RNAi knockdown and *NGT-Gal4 > UASp-tkv.Q253D*.

(A) Ratio (%) of normal, germless, and tumorous ovarioles in the indicated genotypes of 2-5 day-old flies. RNAi knockdown and UASp-tkv.Q253D expression were driven with *UAS-Dcr2* and *NGT-Gal4*. n indicates the total number of ovarioles examined for each genotype. (B) Quantification of GSC-like cell number in germaria of the indicated genotypes from 2-5 day-old flies. Germless ovarioles were excluded from this analysis.

Fig S12. anti-Otu Western blot of dissected ovary lysates.

(A) Western blot of dissected ovary lysates. (B) Western blot of dissected ovary lysates prepared from flies 2-5 hours and 3-7 days post eclosion and co-IP with anti-Sakura. The SDS-PAGE gel was run for a longer duration to better separate the 104 kDa and 98 kDa Otu isoforms. In 2-5 hour

bioRxiv preprint doi: https://doi.org/10.1101/2024.10.04.616675; this version posted June 4, 2025. The copyright holder for this preprint (which was not certified by peer review) is the author/funder, who has granted bioRxiv a license to display the preprint in perpetuity. It is made available under aCC-BY 4.0 International license.

ovaries, the 104 kDa Otu isoform is more abundant, while in 3-7 day ovaries, the 98 kDa isoform predominates. Both Otu isoforms co-IPed with Sakura. The same 3-7 day ovary input and IP samples used for Fig 9B were used for anti-Otu and anti-Alpha-Tubulin Western. The anti-Sakura image is the same as shown in Fig 9B.

Fig S13. Co-immunoprecipitations assay to test interaction between N-terminal fragments of Sakura and Otu in S2 cells

Co-immnuprocepitation assay using beads bound with anti-FLAG antibody followed by Western blotting. S2 cell lysates expressing mCherry-3xHA were used as controls. This is the reciprocal co-immunoprecipitation of Fig 9F.

Fig S14. Depletion of Sakura does not deplete Otu and depletion of Otu does not deplete Sakura

(A) Western blot of dissected ovary lysates. The same samples used for Fig 5B were used for anti-Otu Western. The anti-Sakura and anti-Alpha tubulin images are the same as shown in Fig 5B. (B) The same samples used for Fig 10C were used for anti-Sakura Western. The anti-Otu and anti-Alpha tubulin images are the same as shown in Fig 10C.

Fig S15. In vitro deubiquitination assay.

The mean fluorescence intensity of three replicates was plotted. Error bars (+/-) are standard deviations. Firefly Luciferase was used as a negative control.

bioRxiv preprint doi: https://doi.org/10.1101/2024.10.04.616675; this version posted June 4, 2025. The copyright holder for this preprint (which was not certified by peer review) is the author/funder, who has granted bioRxiv a license to display the preprint in perpetuity. It is made available under aCC-BY 4.0 International license.

Figure S1. Azlan et al.

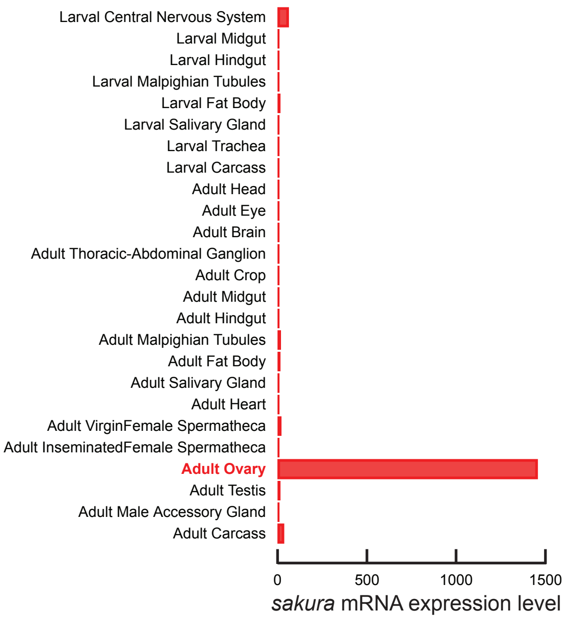

bioRxiv preprint doi: https://doi.org/10.1101/2024.10.04.616675; this version posted June 4, 2025. The copyright holder for this preprint (which was not certified by peer review) is the author/funder, who has granted bioRxiv a license to display the preprint in perpetuity. It is made available under aCC-BY 4.0 International license.

Figure S2: Azlan et al.

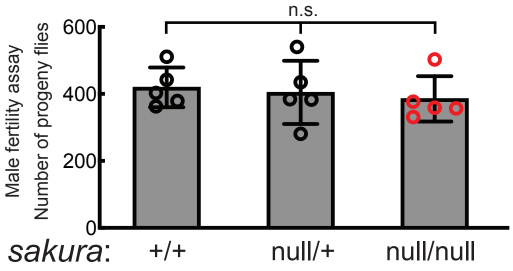

bioRxiv preprint doi: https://doi.org/10.1101/2024.10.04.616675; this version posted June 4, 2025. The copyright holder for this preprint (which was not certified by peer review) is the author/funder, who has granted bioRxiv a license to display the preprint in perpetuity. It is made available under aCC-BY 4.0 International license.

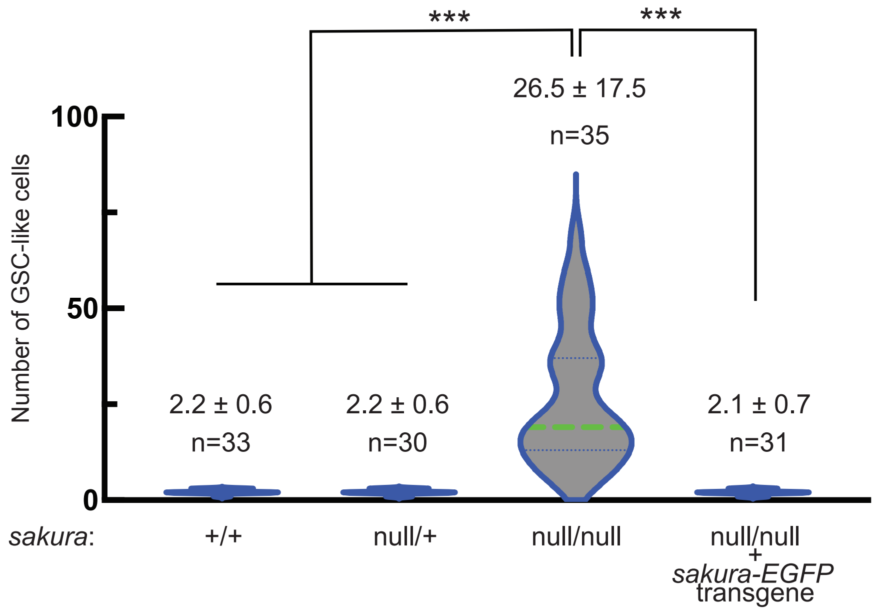

bioRxiv preprint doi: https://doi.org/10.1101/2024.10.04.616675; this version posted June 4, 2025. The copyright holder for this preprint (which was not certified by peer review) is the author/funder, who has granted bioRxiv a license to display the preprint in perpetuity. It is made available under aCC-BY 4.0 International license.

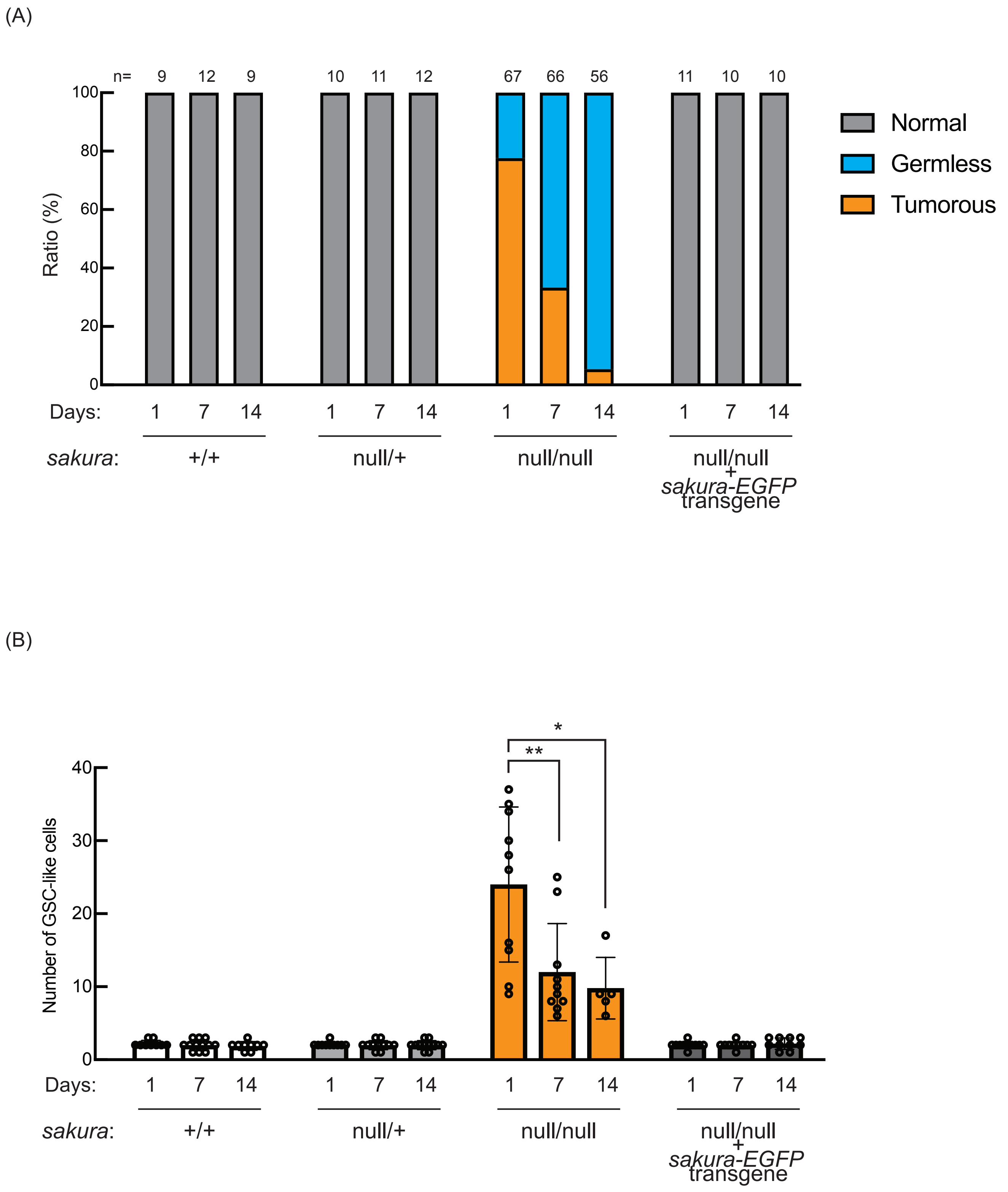

bioRxiv preprint doi: https://doi.org/10.1101/2024.10.04.616675; this version posted June 4, 2025. The copyright holder for this preprint (which was not certified by peer review) is the author/funder, who has granted bioRxiv a license to display the preprint in perpetuity. It is made available under aCC-BY 4.0 International license.

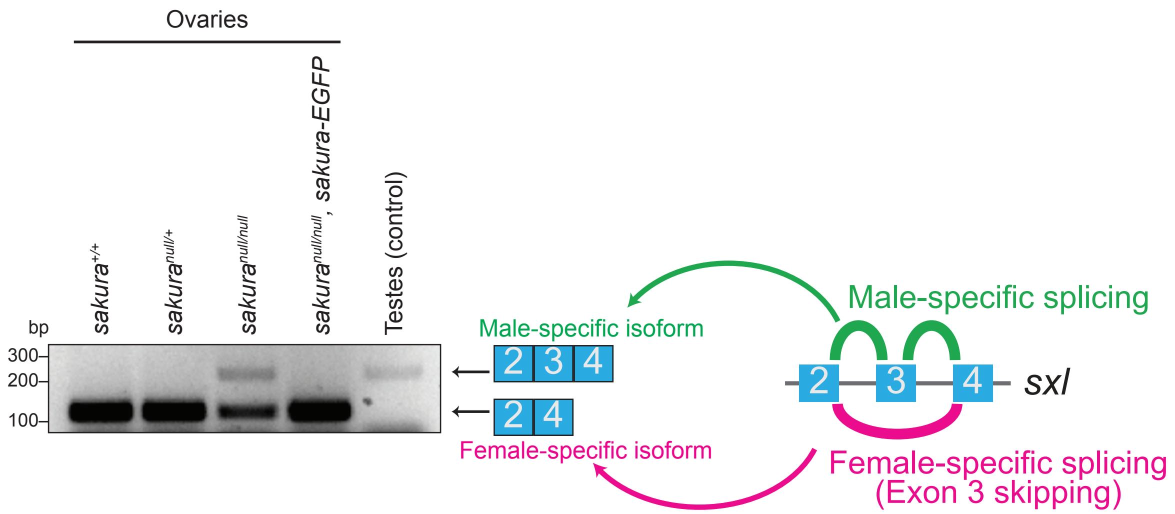

bioRxiv preprint doi: https://doi.org/10.1101/2024.10.04.616675; this version posted June 4, 2025. The copyright holder for this preprint (which was not certified by peer review) is the author/funder, who has granted bioRxiv a license to display the preprint in perpetuity. It is made available under aCC-BY 4.0 International license.

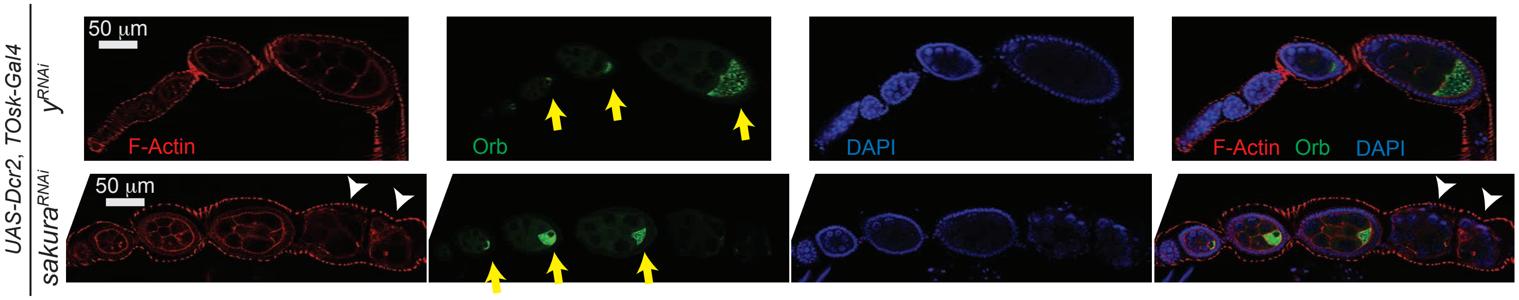

bioRxiv preprint doi: https://doi.org/10.1101/2024.10.04.616675; this version posted June 4, 2025. The copyright holder for this preprint (which was not certified by peer review) is the author/funder, who has granted bioRxiv a license to display the preprint in perpetuity. It is made available under aCC-BY 4.0 International license.

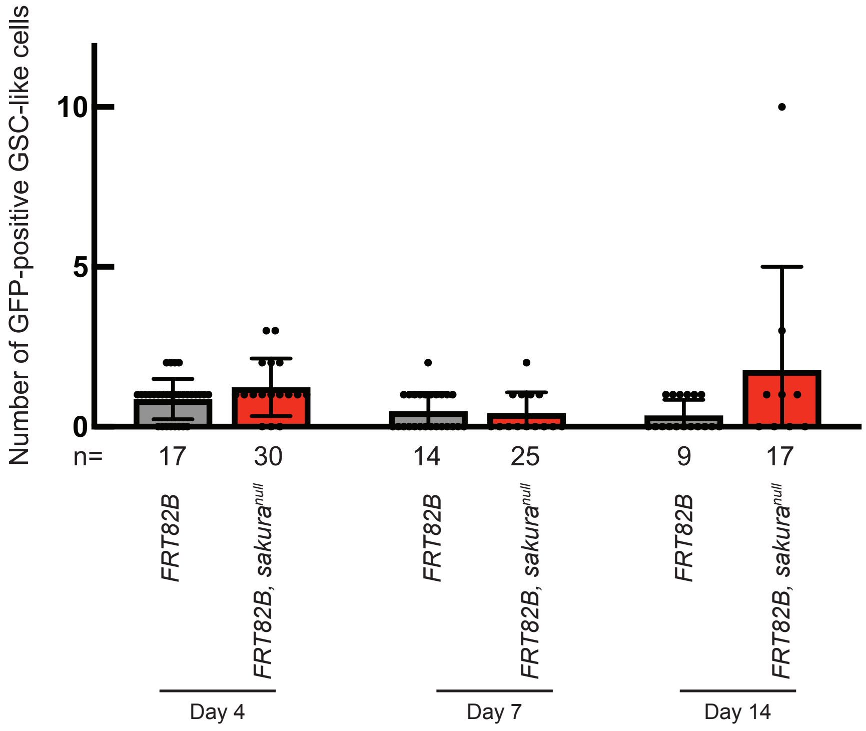

bioRxiv preprint doi: https://doi.org/10.1101/2024.10.04.616675; this version posted June 4, 2025. The copyright holder for this preprint (which was not certified by peer review) is the author/funder, who has granted bioRxiv a license to display the preprint in perpetuity. It is made available under aCC-BY 4.0 International license.

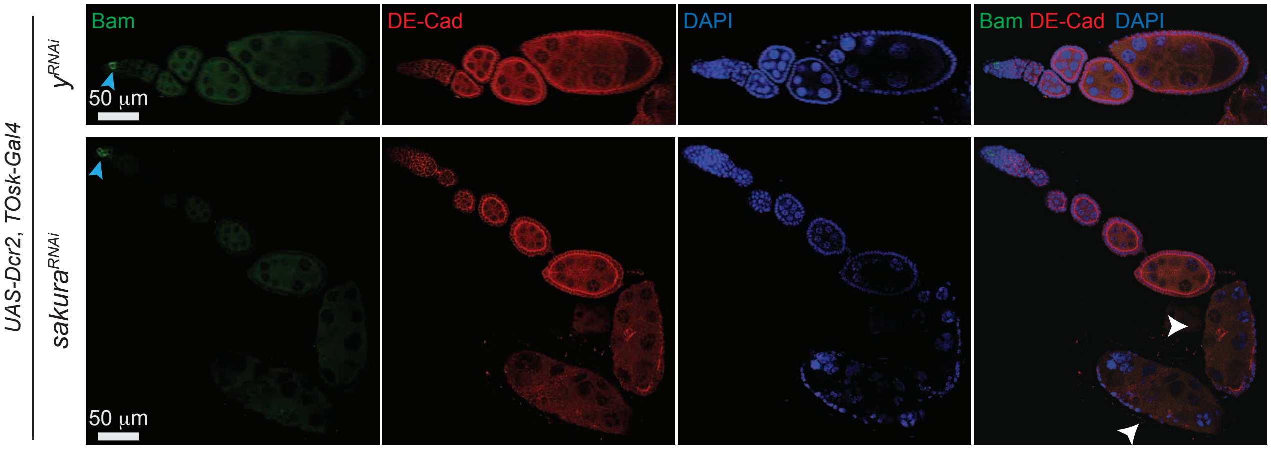

bioRxiv preprint doi: https://doi.org/10.1101/2024.10.04.616675; this version posted June 4, 2025. The copyright holder for this preprint (which was not certified by peer review) is the author/funder, who has granted bioRxiv a license to display the preprint in perpetuity. It is made available under aCC-BY 4.0 International license.

(A)

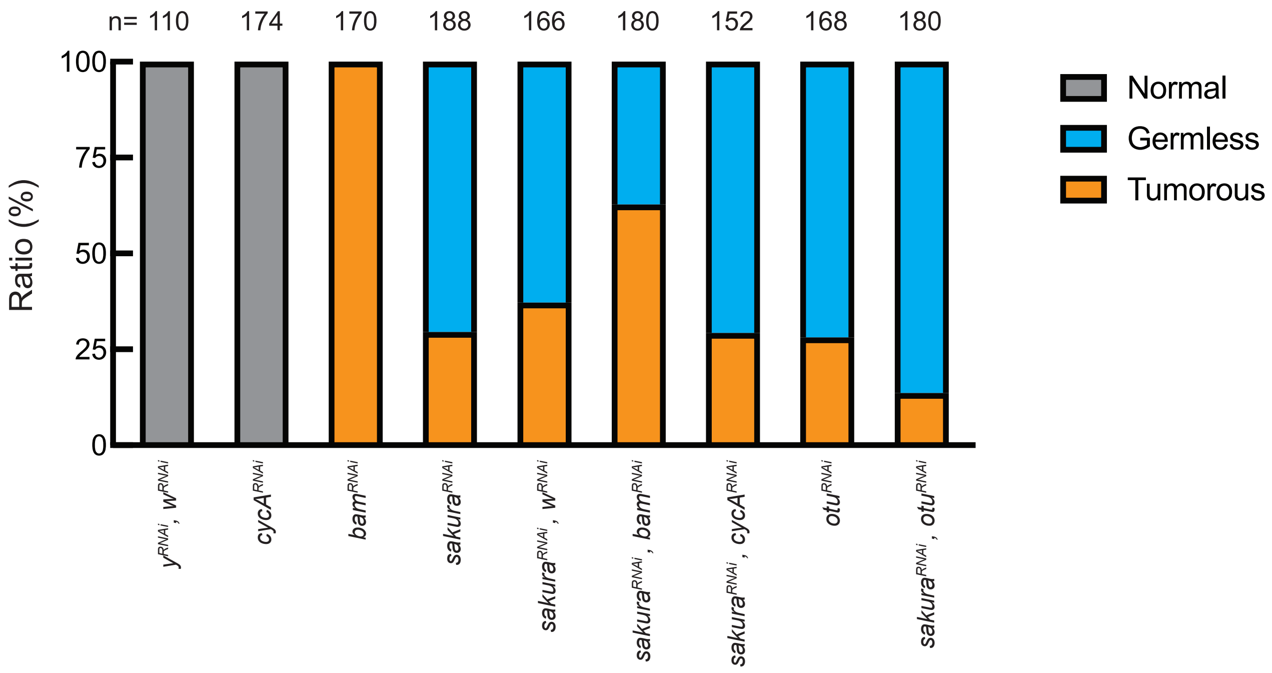

(B)

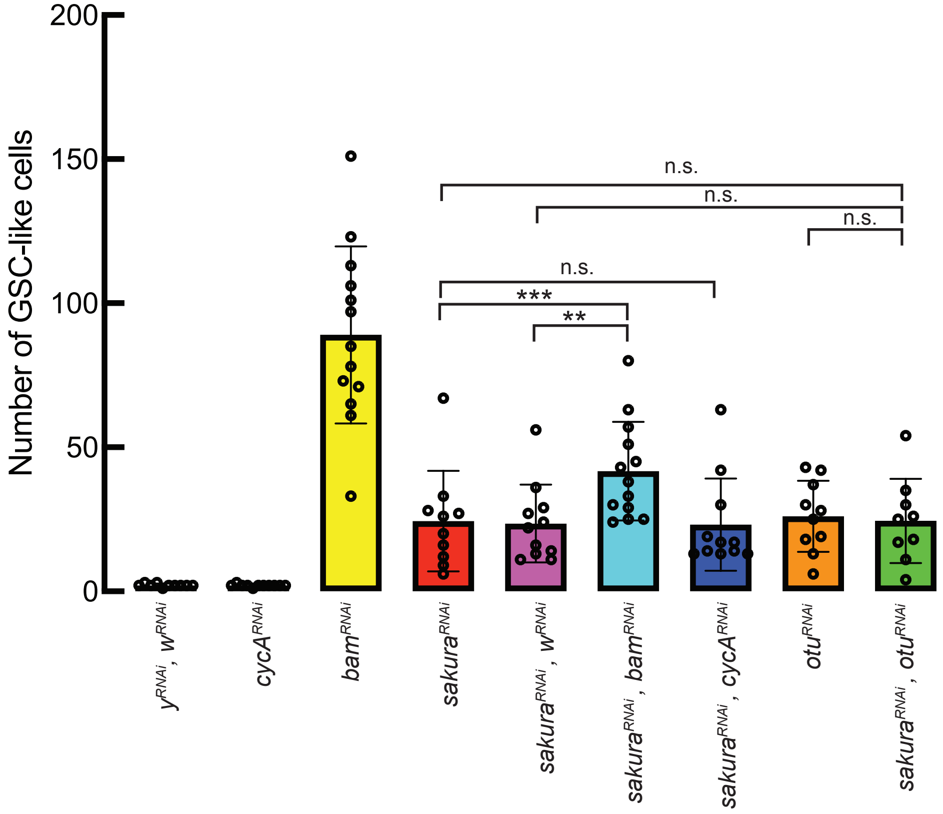

bioRxiv preprint doi: https://doi.org/10.1101/2024.10.04.616675; this version posted June 4, 2025. The copyright holder for this preprint (which was not certified by peer review) is the author/funder, who has granted bioRxiv a license to display the preprint in perpetuity. It is made available under aCC-BY 4.0 International license.

Figure S10. Azlan et al.

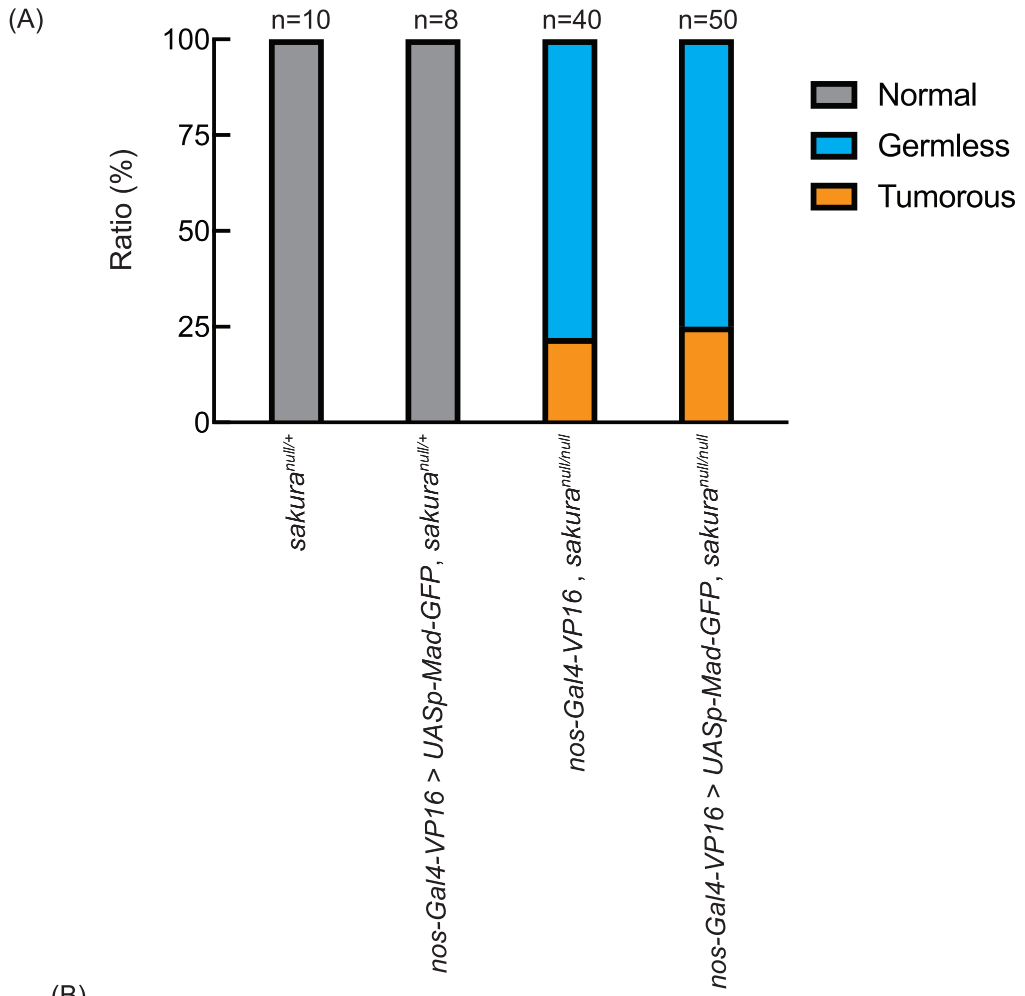

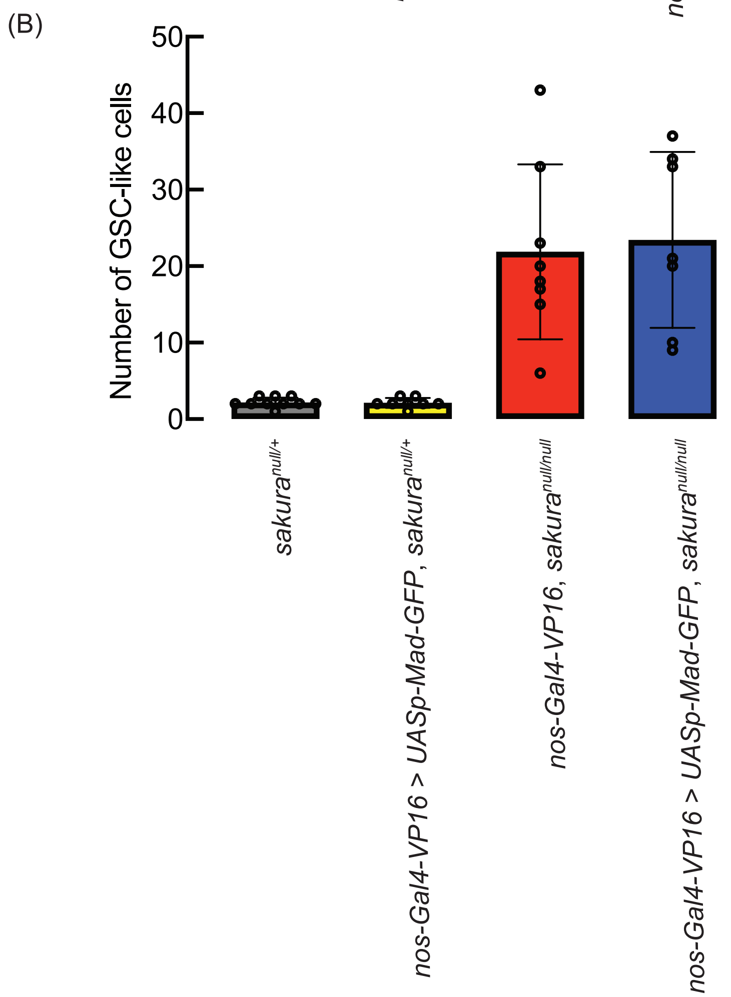

bioRxiv preprint doi: https://doi.org/10.1101/2024.10.04.616675; this version posted June 4, 2025. The copyright holder for this preprint (which was not certified by peer review) is the author/funder, who has granted bioRxiv a license to display the preprint in perpetuity. It is made available under aCC-BY 4.0 International license.

Figure S11. Azlan et al.

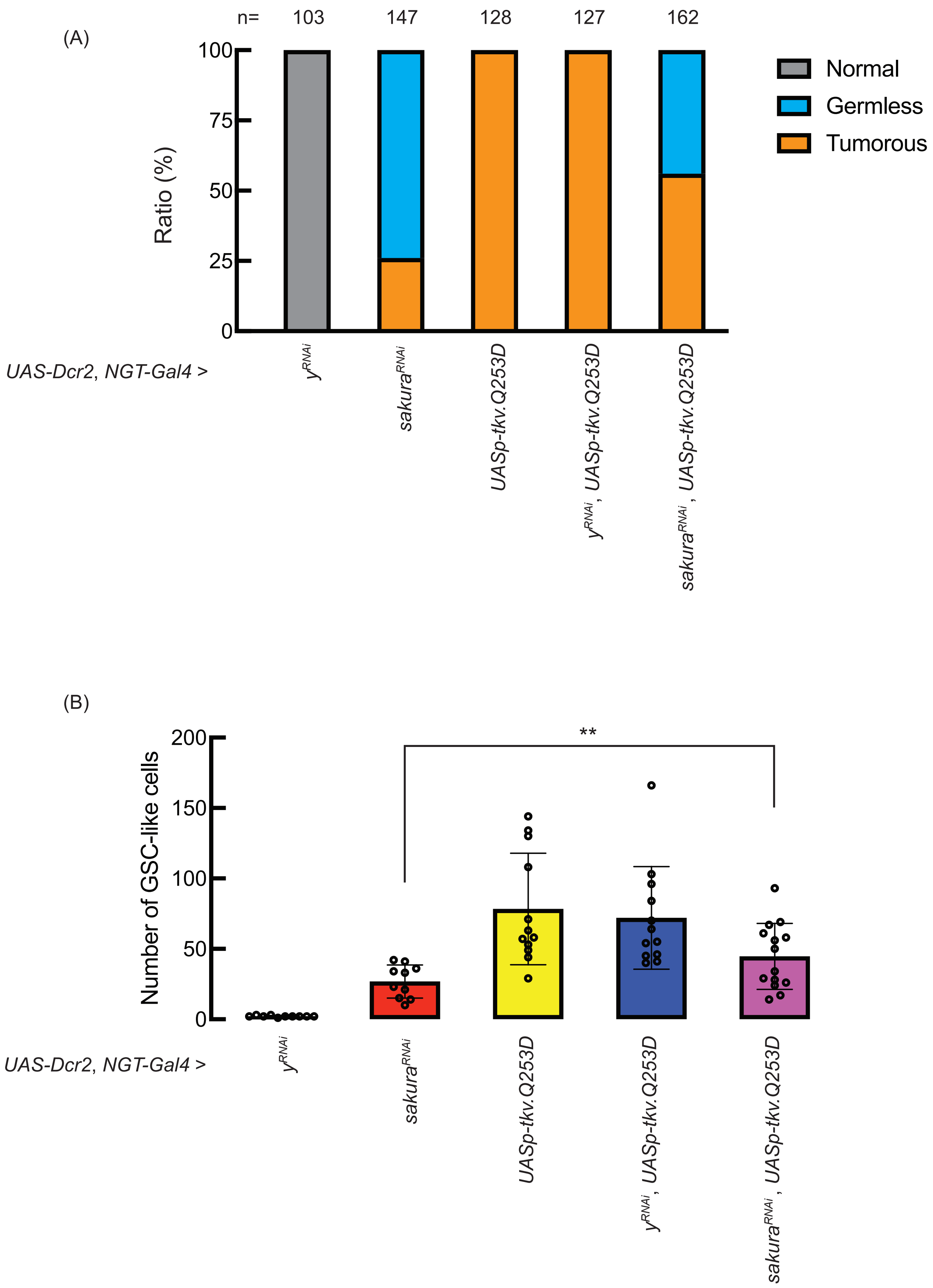

bioRxiv preprint doi: https://doi.org/10.1101/2024.10.04.616675; this version posted June 4, 2025. The copyright holder for this preprint (which was not certified by peer review) is the author/funder, who has granted bioRxiv a license to display the preprint in perpetuity. It is made available under aCC-BY 4.0 International license.

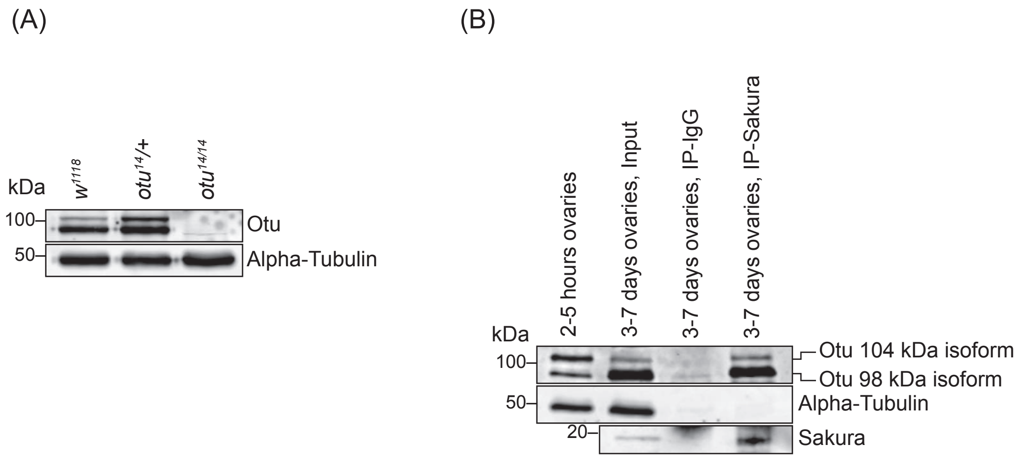

bioRxiv preprint doi: https://doi.org/10.1101/2024.10.04.616675; this version posted June 4, 2025. The copyright holder for this preprint (which was not certified by peer review) is the author/funder, who has granted bioRxiv a license to display the preprint in perpetuity. It is made available under aCC-BY 4.0 International license.

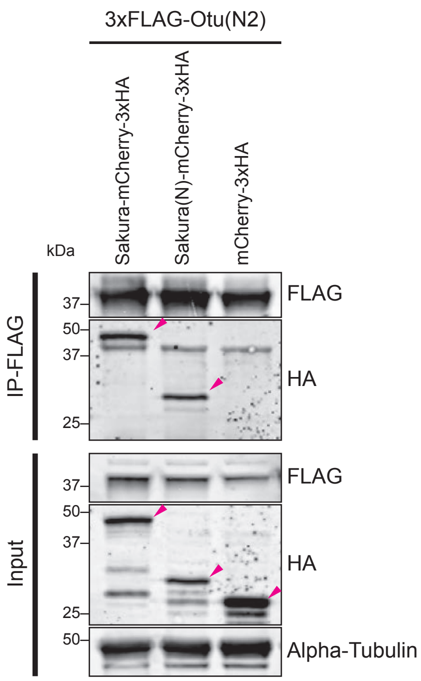

bioRxiv preprint doi: https://doi.org/10.1101/2024.10.04.616675; this version posted June 4, 2025. The copyright holder for this preprint (which was not certified by peer review) is the author/funder, who has granted bioRxiv a license to display the preprint in perpetuity. It is made available under aCC-BY 4.0 International license.

Figure S14. Azlan et al.

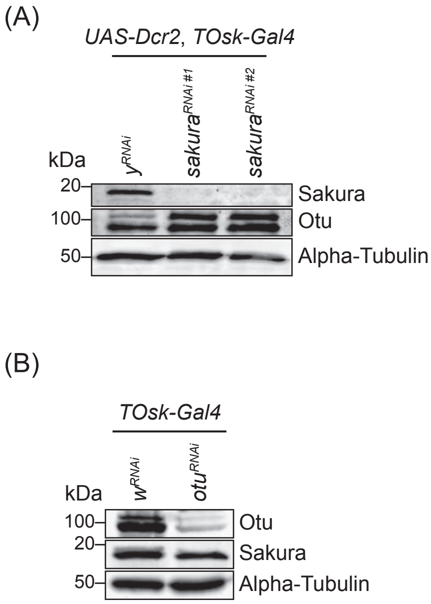

bioRxiv preprint doi: https://doi.org/10.1101/2024.10.04.616675; this version posted June 4, 2025. The copyright holder for this preprint (which was not certified by peer review) is the author/funder, who has granted bioRxiv a license to display the preprint in perpetuity. It is made available under aCC-BY 4.0 International license.

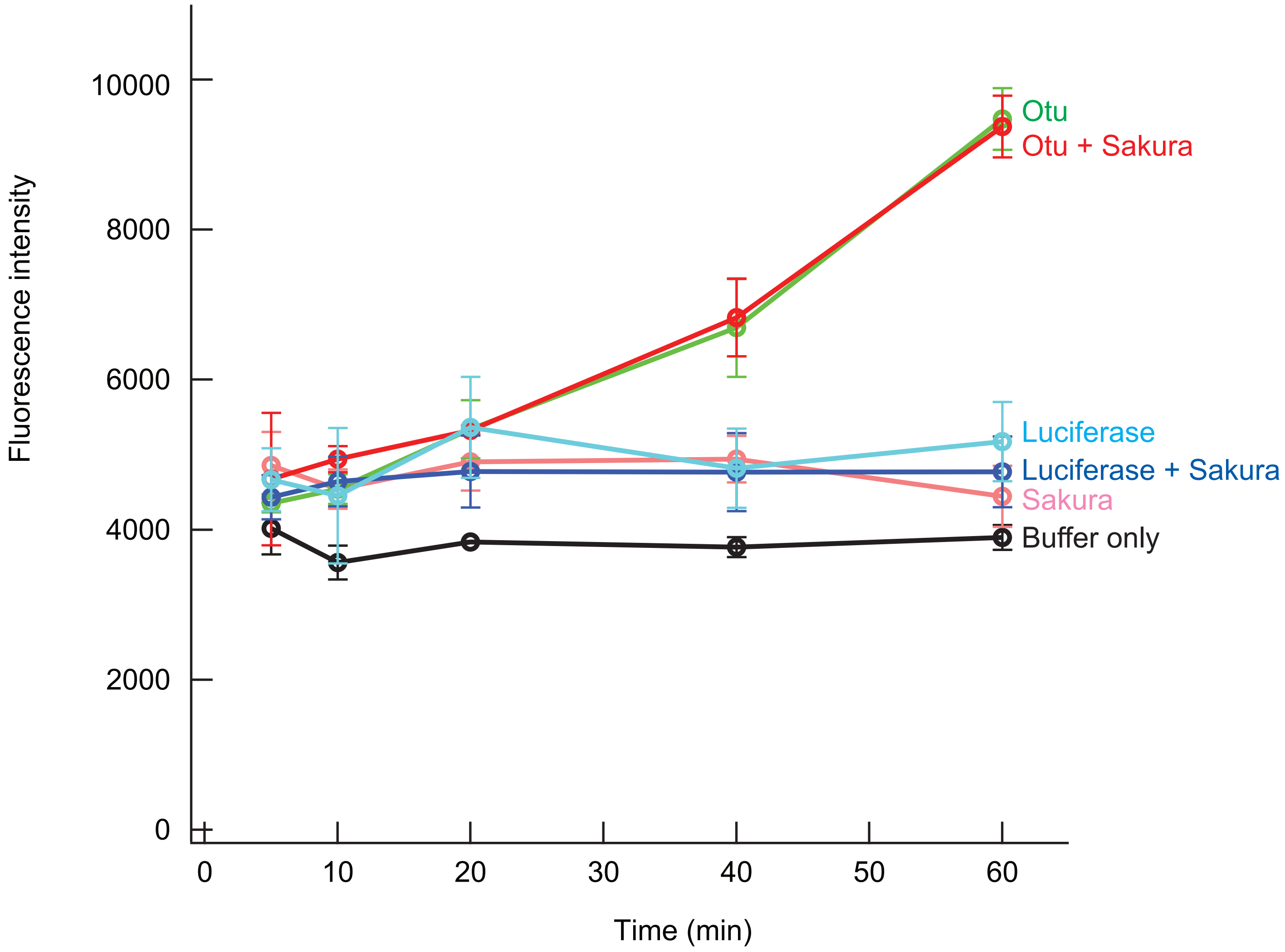

Supplement: Supplement 1 [file NIHPP2024.10.04.616675v4-supplement-1.pdf]
